# Molecular Signaling and Metabolic Responses during the Interaction between Human Keratinocytes (HaCaT) and the Dermatophyte *Trichophyton rubrum*

**DOI:** 10.3390/jof10010072

**Published:** 2024-01-16

**Authors:** Monise Fazolin Petrucelli, Leonardo Martins-Santana, Antonio Rossi, Nilce Maria Martinez-Rossi

**Affiliations:** Department of Genetics, Ribeirão Preto Medical School, University of São Paulo, Ribeirão Preto 14049-900, SP, Brazil; mofazolin@gmail.com (M.F.P.); leonardo.lms95@gmail.com (L.M.-S.); anrossi@usp.br (A.R.)

**Keywords:** immune response, pathogen–host interaction, cell wall, glucose, lactate, PRRs, *Trichophyton rubrum*, keratinocytes

## Abstract

*Trichophyton rubrum* is the leading causative agent of dermatophytosis worldwide. Keratinocytes are the first line of defense that drives an immune response against fungal invasion. Host-specific pattern recognition receptors (PRRs) recognize pathogen-associated molecular patterns (PAMPs) to trigger immunological pathways. Fungal cell wall components are the primary sources of fungal PAMPs, and some pathogens increase cell wall rearrangement to evade the immune system. Glycolysis and enhanced lactate levels are critical for improving host immune responses to fungal infections. Using reverse transcription–quantitative polymerase chain reaction (RT-qPCR), we evaluated the transcriptional responses of human genes involved in fungal recognition and glycolytic metabolism and fungal cell-wall-related genes in a co-culture model of human keratinocytes with *T. rubrum*. We observed the upregulation of several Toll-like receptors (TLRs), NOD-like receptors (NLRs), and glycolytic genes. Complementarily, we measured intra- and extracellular glucose levels and the increase in lactate production in the co-culture supernatant. We noted a distinct transcriptional regulation pattern of fungal cell-wall-related genes from fungal growth on keratin as the primary carbon source compared to co-culture with human keratinocytes. Our results showed new insights into the transcriptional adaptation of keratinocytes, particularly in regulating genes involved in sensing and metabolic processes, during the interaction with *T. rubrum*.

## 1. Introduction

Cutaneous infections caused by dermatophytes are among the most prevalent types of mycoses, affecting over 25% of the global population. Disorders such as immunosuppression and diabetes can aggravate the development of dermatophytosis [1,2]. Among dermatophyte species, *Trichophyton rubrum* is responsible for over 60% of all dermatophytosis in humans, affecting keratinized tissues, mainly the skin and nails [3].

During dermatophyte–host interactions, keratinized tissue is the principal nutrient source for fungal growth [4]. Coordinated steps of fungal keratinolytic protease secretion, such as hydrolases (endo- and exopeptidases), accompanied by pH sensing and metabolic adaptation, enable the pathogen to adhere to the host tissue and utilize the available nutrients for survival and growth [3,5,6,7]. Furthermore, extracellular pH changes are crucial for regulating gene expression as a fungal strategy for colonization, development, and maintenance in the host [8].

Keratinocytes are the most abundant cell type in the epidermis. They play a pivotal role in triggering an innate immune response during infection. As an efficient and protective barrier against microbial attacks and external factor damage, keratinocytes are the first to contact fungi during invasion [9]. In addition, these cells express various Pattern Recognition Receptors (PRRs), including Toll-like receptors (TLRs), C-type lectin receptors (CLRs), and nucleotide-binding oligomerization domain-like receptors (NOD-like receptors) [10,11]. These proteins are capable of recognizing molecules frequently found in pathogens, called Pathogen Associated Molecular Patterns (PAMPs), and trigger the activation of distinct signaling pathways, producing inflammatory cytokines, chemokines, and antimicrobial peptide molecules, which promote the activation and recruitment of immune cells to the site of infection [11].

Glucose is critical for supporting immune cells in the host defense against fungal infections. Maintaining glucose homeostasis is a pivotal factor during bacterial and viral infections and potentially in the context of fungal infections. This effect arises from the central role of glucose metabolism in driving the protective immune response, primarily through its influence on cytokine production [12]. Increased glycolysis, followed by reduced mitochondrial oxidative phosphorylation, leads to an aerobic glycolytic scenario known as the Warburg effect, wherein glucose drives lactate generation without further oxidation by mitochondria. Lactate generated during the Warburg effect can act as a signaling molecule that orchestrates an effective immune response [13]. Keratinocytes alter their metabolic pathways to achieve the Warburg effect to defend against bacterial [14] or viral infections [15]. However, the molecular mechanism of aerobic glycolysis upon fungal stimulation, especially in dermatophytes, remains unclear.

Sensing the host milieu, pathogenic fungi, such as *T. rubrum*, modulate their cell wall components as a defense mechanism to escape host immunity and PRR recognition [16]. Fungal cell walls play an essential role in various biological functions, including the control of cellular permeability and protection from osmotic and mechanical stresses. Furthermore, some cell wall components such as chitin, glucans, and mannans are immunogenic. Recognizing PAMPs through host PRRs is crucial in stimulating cellular and humoral immune responses during infection [17].

Understanding the host cell response at the initial stage of fungal infection is critical. We know little about the cellular mechanisms activated in response to dermatophyte infections. The availability of nutrients during infection is an essential metabolic factor determining how host cells protect themselves from energy depletion while preventing pathogen invasion. Thus, this study aimed to assess *T. rubrum*–host interactions by analyzing host PRRs and fungal enzymes involved in cell wall remodeling at different time points of interaction. Additionally, we investigated how keratinocytes modulate the expression of genes associated with glycolytic metabolism to augment their effector response.

## 2. Materials and Methods

### 2.1. Strains, Media, and Growth Conditions

*Trichophyton rubrum* strain (CBS118892), isolated from a male patient with onychomycosis (Westerdijk Fungal Biodiversity Institute, Utrecht, The Netherlands), was grown on solid malt extract agar medium (2% glucose, 2% malt extract, 0.1% peptone, 2% agar, pH 5.7) for 15 days in an incubator at 28 °C. Approximately 1 × 10^6^ conidia were inoculated in 100 mL of Sabouraud dextrose broth, followed by incubation at 28 °C for 96 h with agitation.

The resulting mycelia were washed with sterile water and transferred into 100 mL minimal medium [18] supplemented with 70 mM sodium nitrate (Sigma-Aldrich, St. Louis, MO, USA) or 0.5% bovine keratin (*m*/*v*). The cultures were then incubated for 24 and 48 h at 28 °C with shaking, with filtered fungal residues from three independent replicates, then quickly frozen, and stored at −80 °C for RNA extraction. Immortalized human keratinocyte HaCaT cells were purchased from Cell Line Service GmbH (Eppelheim, Germany) and cultured in RPMI-1640 medium (Sigma-Aldrich) supplemented with 10% fetal bovine serum at 37 °C in a humidified atmosphere containing 5% CO_2_. Penicillin (100 U/mL) and streptomycin (100 µg/mL) were added to the RPMI-1640 to prevent bacterial contamination.

### 2.2. Co-Culture of Human Keratinocytes and T. rubrum

For the co-culture assay, 2 × 10^5^ HaCaT cells/mL were plated in six-well plates and cultured in RPMI-1640 medium (Sigma Aldrich) supplemented with 5% fetal bovine serum at 37 °C in a humidified atmosphere containing 5% CO_2_ for 24 h until the cells adhered to the plates. To prepare conidial suspensions, plates were flooded with 0.9% sterile NaCl solution, and the samples were filtered through fiberglass. The conidial concentration was estimated using a Neubauer chamber. Approximately 1 × 10^6^ conidia of *T. rubrum* were inoculated into the keratinocyte culture and incubated for 3, 24, or 48 h (Figure S1). Before inoculation, we measured the pH of the cell culture supernatant and after 3, 24, and 48 h in both uninfected keratinocytes and co-culture.

Uninfected cultured keratinocytes and *T. rubrum* conidia grown in RPMI-1640 were used as controls, and the assay was performed three times independently.

### 2.3. RNA Extraction and cDNA Synthesis

For RNA extraction, the fungal cell wall in co-culture experiments was disrupted using a lysis solution containing 20 mg/mL of lysing enzymes from *Trichoderma harzianum* (Sigma-Aldrich), 0.7 M KCl, and 1 M MgSO_4_, pH 6.8, and incubated under gentle shaking for 1 h at 28 °C. After incubation, the samples were centrifugated at 1000× *g* for 10 min, as described previously [19]. Per the manufacturer’s instructions, total RNA was extracted using the Illustra RNAspin Mini RNA Isolation Kit (GE Healthcare, Chicago, IL, USA). After extraction, the RNA concentration and quality were estimated using a NanoDrop ND-100 spectrophotometer (Thermo Fisher Scientific, Waltham, MA, USA).

In subsequent reactions, 1 µg of total RNA was treated with DNAse I (Sigma-Aldrich). Per the manufacturer’s instructions, the High-Capacity cDNA Reverse Transcription Kit (Applied Biosystems, Waltham, MA, USA) was used for cDNA conversion. The cDNAs obtained were analyzed for concentration and purity, suspended in 70 ng/µL dilutions, and stored at −80 °C until the real-time quantitative polymerase chain reaction (RT-qPCR) analysis.

### 2.4. Real-Time Quantitative Polymerase Chain Reaction

Transcriptional quantification was performed using a QuantStudio 3 Real-Time PCR System (Applied Biosystems). Per the manufacturer’s instructions, the reactions were carried out using a Power SYBR™ Green PCR Master Mix (Applied Biosystems) with a ROX dye as a fluorescent normalizer [20]. For relative expression analysis, the 2^−ΔΔCt^ method [21] was used to determine the expression of human GAPDH [22], β-actin [23], and *T. rubrum gapdh* [5] and *rpb2* [24] genes as endogenous controls for expression normalization. Uninfected HaCaT cells or fungal cells cultured in RPMI without HaCaT cells were used as co-culture controls for human and fungal gene analysis. Glucose growth conditions were used to analyze gene expression in different carbon sources during fungal growth. The concentration of each primer was standardized for reaction efficiencies between 90% and 110%. The primers used for the RT-qPCR analysis were listed in Supplementary Table S1 and the results were presented as the mean relative expression values from three independent replicates with standard deviations.

### 2.5. Glucose Quantification

The intracellular and extracellular glucose levels in keratinocytes were quantified during co-culture. After 3 h, 24 h, and 48 h of co-culture, the cell culture supernatant was collected and stored at −80 °C for subsequent glucose measurements. The adherent cells were trypsinized, washed with Phosphate-Buffered Saline (PBS 1X, pH 7.4), and then disrupted using 350 μL of a lysis buffer solution (50 mM Tris-HCl, pH 7.4, 150 mM NaCl, 1 mM EDTA, 1% (*v*/*v*) Triton X-100) on ice. Subsequently, the lysate was centrifuged at 10,000× *g* for 15 min at 4 °C to eliminate insoluble debris, including fungal cells in the co-culture. We stored the resulting soluble fraction at −80 °C for subsequent intracellular glucose analysis.

Per the manufacturer’s instructions, the glucose levels were determined using the Glucose PAP Liquiform Kit (Labtest Diagnóstica, Lagoa Santa, MG, Brazil). Then, 20 µL of either sample or cell culture supernatant was mixed with 2 mL of the Glucose PAP liquiform color reagent. The absorbance was measured at 505 nm using an Evolution™ 201 UV-Visible Spectrophotometer (Thermo Fisher Scientific). Glucose concentrations (mg/mL) in the samples and cell supernatants were determined using standard curves generated from serial dilutions of glucose.

For the absorbance measurement to determine the glucose content in the cell culture supernatant and intracellular glucose, RPMI-1640 5% fetal bovine serum and cell lysis solution were used to obtain baseline absorbance readings. Uninfected HaCaT cells were used as a control to evaluate the differences in the glucose content between HaCaT cells during co-culture. Three independent biological replicates were used in each experiment.

### 2.6. Lactate Quantification

The lactate content in the cell culture supernatant was assessed after 3 h, 24 h, and 48 h of co-culture using the Lactate Enzymatic Kit (Labtest Diagnóstica). Per the manufacturer’s instructions, 20 µL of cell culture supernatant was mixed with 2 mL of Lactate Enzymatic color reagent. Absorbance readings were taken at 550 nm using an Evolution™ 201 UV-Visible Spectrophotometer (Thermo Fisher Scientific).

The lactate concentration (mg/mL) was determined in the cell culture supernatant using standard curves generated from serial dilutions of lithium lactate. We used RPMI-1640 5% fetal bovine serum to obtain a baseline absorbance reading for the absorbance measurement of the lactate content in the cell culture supernatant. Uninfected HaCaT and fungal cell culture supernatants were used as a control to evaluate the differences in the lactate content during co-culture. Three independent biological replicates were used in each experiment.

### 2.7. Enzymatic Dosage of Lactate Dehydrogenase (LDH)

Following the manufacturer’s instructions, the LDH activity intracellularly and in the cell culture supernatant was assessed using a Lactate Dehydrogenase activity assay kit (Sigma-Aldrich). After 3 h, 24 h, and 48 h of co-culture, the cell culture supernatant was collected and preserved at −80 °C for subsequent LDH activity measurements. Adherent cells were trypsinized and washed with Phosphate-Buffered Saline. The cellular pellets were resuspended in 350 µL of cold LDH Assay Buffer. Subsequently, this suspension was centrifuged at 10,000× *g* for 15 min at 4 °C to eliminate cellular debris, including fungal cells in the co-culture, and the resulting soluble fraction was stored at −80 °C for later LDH activity assessments.

LDH activity was ascertained based on NADH oxidation, and absorbance readings were recorded at 450 nm using a Multiscan FC microplate reader (Thermo Fisher Scientific). Uninfected HaCaT cells were used as controls. For quantifying LDH activity in the cell supernatant, we used RPMI-1640 containing 5% fetal bovine serum as a negative control. Simultaneously, LDH Assay Buffer was utilized as a negative control for intracellular LDH activity.

Three independent biological replicates were performed for each experimental condition.

### 2.8. Statistical Analysis

Statistical analysis was performed using the unpaired *t*-test and statistical significance determined using the Holm–Sidak method considering * *p* < 0.05, ** *p* < 0.01, *** *p* < 0.001, and **** *p* < 0.0001. GraphPad Prism software (version 8.2; GraphPad Software, San Diego, CA, USA) was used to generate statistical analyses and graphs.

## 3. Results

### 3.1. Transcriptional Response of Keratinocyte PRR Genes

**Figure 1:**
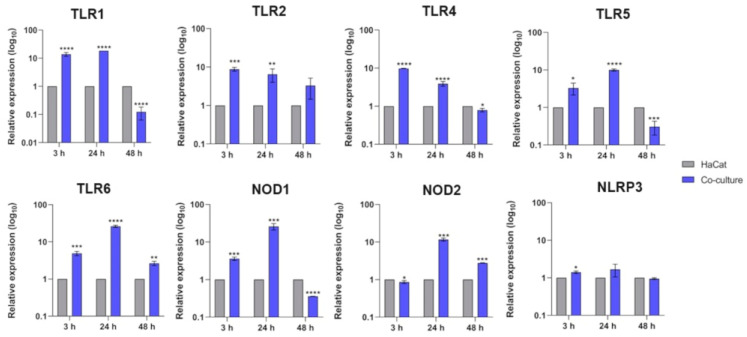
Relative expression analyses of human keratinocytes’ (HaCaT) PRRs (TLRs and NLRs) during co-culture with *T. rubrum*. Statistical significance was determined using an unpaired Student’s *t*-test with Holm–Sidak correction for multiple testing considering * *p* < 0.05, ** *p* < 0.01, *** *p* < 0.001, and **** *p* < 0.0001.

We evaluated the transcriptional modulation of several PRR genes, including TLR family members 1–9, NLRs, and C-type lectin receptors. In the TLR family, we observed the upregulation of TLR1, TLR2, TLR4, TLR5, and TLR6 transcripts at 3 h and 24 h post-interaction. Similarly, transcripts of NLR family members, NOD1 and NLRP3 (NLR family pyrin domain-containing 3), were upregulated at 3 h, but only NOD1 and NOD2 were positively modulated at 24 h (Figure 1). After 48 h, we observed a decrease in the transcript levels of both TLR and NLR family members. Under these conditions, we did not observe any significant transcriptional response for the C-type lectin receptor Dectin-1.

### 3.2. Overexpression of Human MTOR, HIF-1α, and the Glycolytic Genes (PDHA, GLUT1, and LDHA)

**Figure 2:**
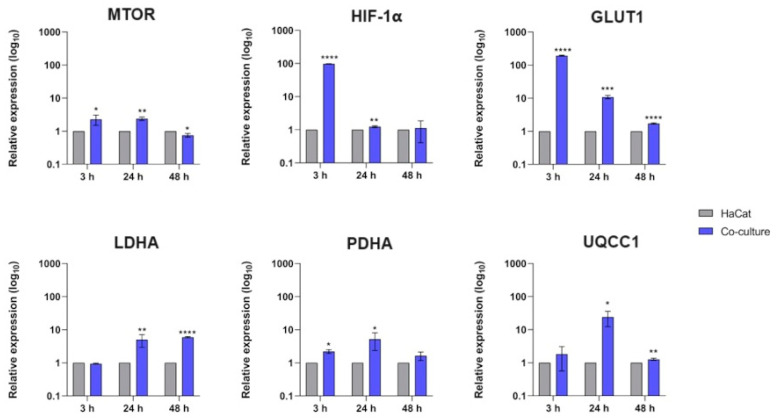
Relative expression analyses of human genes involved in nutrient signaling and glycolytic metabolism during co-culture with *T. rubrum*. Statistical significance was determined using an unpaired Student’s *t*-test with Holm–Sidak correction for multiple testing considering * *p* < 0.05, ** *p* < 0.01, *** *p* < 0.001, and **** *p* < 0.0001.

We evaluated the transcriptional response of crucial genes involved in nutrient signaling, such as MTOR and HIF-1α, and the glycolytic genes PDHA (pyruvate dehydrogenase), GLUT1 (glucose transporter 1), and LDHA (lactate dehydrogenase). Furthermore, we analyzed the transcript levels of UQCC1 (ubiquinol-cytochrome C reductase complex assembly factor 1) involved in mitochondrial oxidative phosphorylation (Figure 2). These data show upregulation in genes coding members of the mTOR-HIF-1α pathway after 3 h and 24 h of co-culture. We also detected high levels of the glycolytic genes GLUT1 and PDHA. We also observed an upregulation of LDHA at 24 h, followed by a subsequent decrease in the transcriptional level of UQCC1 at 48 h.

### 3.3. Intracellular Glucose Levels Increase in HaCaT Cells

**Figure 3:**
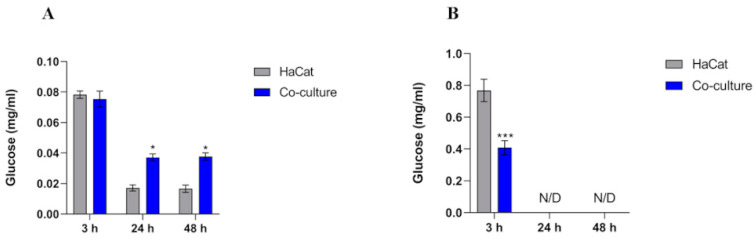
Intracellular (**A**) and extracellular (**B**) glucose content comparison between HaCaT keratinocytes (control) and HaCaT keratinocytes co-cultured with *T. rubrum*. Statistical significance was determined using an unpaired Student’s *t*-test with Holm–Sidak correction for multiple testing considering * *p* < 0.05 and *** *p* < 0.001. N/D means the absence of significant glucose levels detected in the control and co-culture conditions.

The intracellular glucose content showed higher levels after 3 h of co-culture (Figure 3A). We did not observe a statistically significant difference in glucose levels throughout the co-culture conditions compared with the control. Conversely, a reduction in glucose content was observed at 24 and 48 h of co-culture relative to the initial 3 h of fungus–host interaction. HaCaT cells in contact with fungi showed augmented intracellular glucose levels. Significant extracellular glucose levels were detected only after 3 h of incubation in the control (uninfected keratinocytes) and co-culture (Figure 3B). In contrast to the intracellular glucose measurements, extracellular glucose levels were higher in the control group than in the co-culture.

### 3.4. Over-Induction of Lactate Levels in the Cell Supernatant

**Figure 4:**
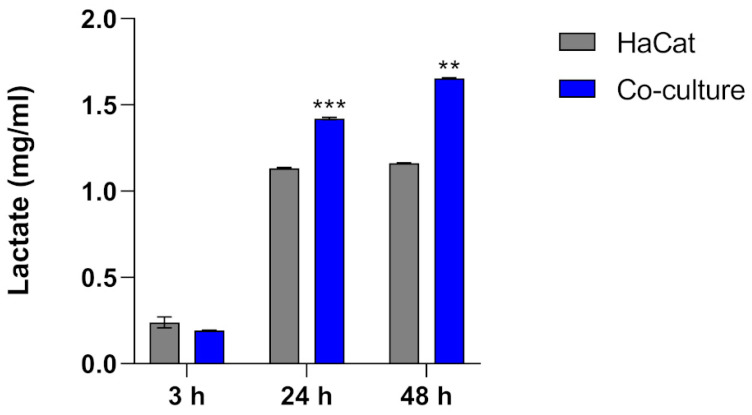
Extracellular lactate content in HaCaT keratinocytes (control) and HaCaT keratinocytes co-cultured with *T. rubrum.* Statistical significance was determined using an unpaired Student’s *t*-test with Holm–Sidak correction for multiple testing considering ** *p* < 0.01 and *** *p* < 0.001.

In addition to intra- and extracellular glucose levels, we assessed the lactate levels in the cell culture supernatant. The results demonstrated a significant increase in lactate levels during co-culture at 24 and 48 h compared to uninfected HaCaT cells (Figure 4).

### 3.5. Intracellular LDH Activity Decreases in Co-Culture, Whereas the Extracellular Activity Boosts in 24 h

**Figure 5:**
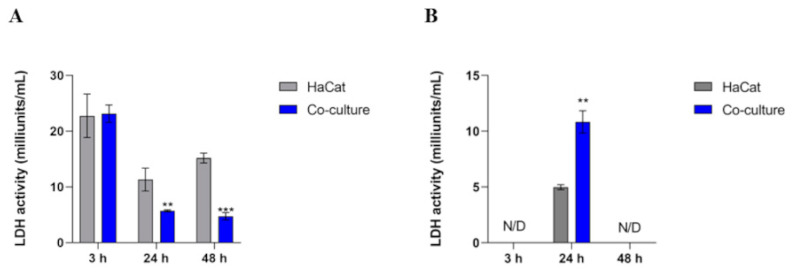
Intracellular (A) and extracellular (B) LDH activity measurements in HaCaT keratinocytes (control) and HaCaT keratinocytes co-cultured with *T. rubrum*. Statistical significance was determined using an unpaired Student’s *t*-test with Holm–Sidak correction for multiple testing considering ** p < 0.01 and *** p < 0.001. N/D means the absence of significant LDH activity detected in the control and co-culture conditions.

We measured the intracellular and extracellular lactate dehydrogenase (LDH) activities of co-cultured cells. Within the first 3 h, higher intracellular LDH activity was observed in the control and co-culture conditions. However, enzyme activity diminished in both scenarios over 24 and 48 h. Notably, this reduction was more pronounced in the co-cultured samples compared to the uninfected keratinocytes (Figure 5A). Regarding the analysis of LDH activity in the cell culture supernatant, we detected enzymatic activity only at the 24 h mark. Notably, there was a more significant increase in LDH activity in the co-culture than in the control, as shown in Figure 5B.

We also monitored the pH of the cell culture supernatant during the co-culture assay to assess any pH changes that could interfere with LDH enzymatic activity (Table 1). The pH of the cell culture supernatant before fungal inoculation was 7.0.

### 3.6. Transcript Levels of Cell-Wall-Related Genes Are Boosted during Fungal Growth in Keratin but Show Different Regulation Patterns in Co-Culture

Fungal chitins and β-glucans are essential sources of fungal PAMPs for host recognition, and trigger immunological responses. In a mimicked infection-like scenario, we monitored transcript levels of genes encoding crucial enzymes for fungal cell wall remodeling during co-culture and fungal growth in different carbon sources. The genes included chitin synthase and β-glucan synthase.

**Figure 6:**
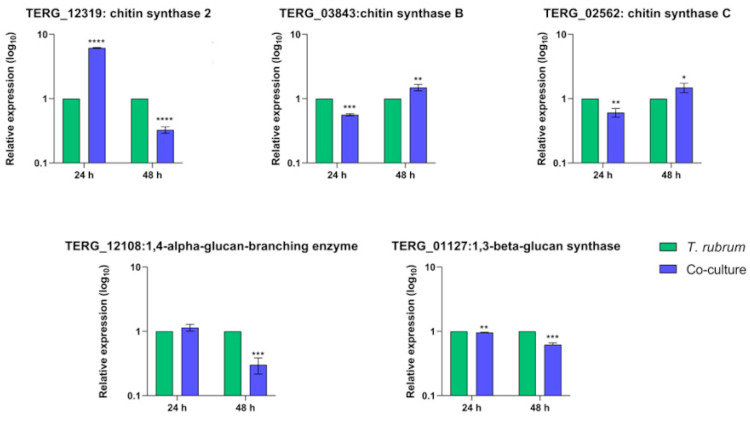
Relative expression analysis of fungal cell wall genes during co-culture with keratinocytes. Expression levels for each condition are compared to the control (*T. rubrum* grown in RPMI-1640 medium without HaCaT cells). Statistical significance was determined using an unpaired Student’s *t*-test with Holm–Sidak correction for multiple testing considering * *p* < 0.05, ** *p* < 0.01, *** *p* < 0.001, and **** *p* < 0.0001.

As shown in Figure 6, the transcriptional level of the gene encoding the beta-glucan synthase was downregulated at 24 h (TERG_01127) and 48 h (TERG_01127 and TERG_12108) of co-culture. Curiously, transcripts of chitin synthase 2 (TERG_12319) showed different patterns of expression compared to the transcripts of chitin synthases B (TERG_03843) and C (TERG_02562). We observed a negative modulation of TERG_03843 and TERG_02562 transcript levels at 24 h post-interaction. We detected the opposite effect after 48 h of co-culture. In addition, our results showed that the TERG_12319 transcript level pattern was the opposite, which suggests a different mechanism of transcriptional regulation of chitin synthase enzymes during infection.

**Figure 7:**
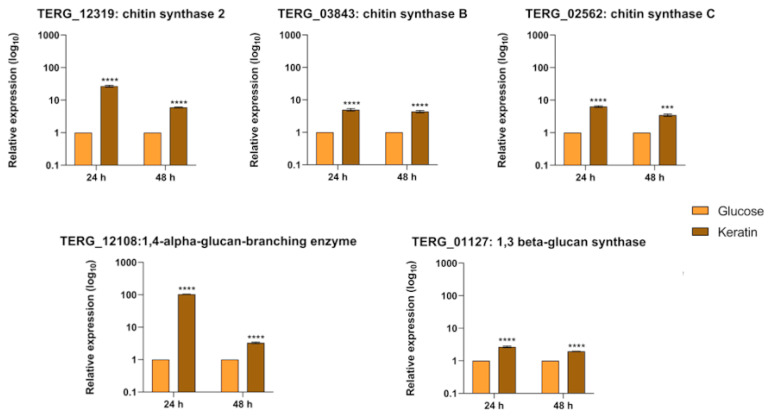
Relative expression analysis of fungal cell-wall-related genes during switching from glucose to keratin. Expression levels for each condition are compared to the control (*T. rubrum* grown in glucose medium). Statistical significance was determined using an unpaired Student’s *t*-test with Holm–Sidak correction for multiple testing considering *** *p* < 0.001 and **** *p* < 0.0001.

Regarding growth in different carbon sources, we observed increased transcriptional levels of fungal cell-wall-related genes at 24 h and 48 h after switching from glucose to keratin media (Figure 7). Compared to the co-culture, these genes were induced during fungal growth in a keratin-containing medium at all time points, indicating a different balance in transcriptional regulation.

## 4. Discussion

An accurate innate immune response is the first line of host defense against fungal infections [10], with keratinocytes playing an essential role in this immune response. By sensing environmental conditions, keratinocytes can trigger a series of molecular events that result in cytokine, chemokine, and antimicrobial peptide production, as well as in the recruitment of phagocytes to the site of infection. PAMPs of fungal pathogens are recognized by several PRRs, such as CLRs, TLRs, and NLRs. Among PRRs, TLRs are crucial for host defense [10,11,25,26].

Previous studies showed that chitin and mannans, relevant fungal PAMPs, are recognized by TLR2 and TLR4, respectively [27,28]. It has also been reported that upon *T. rubrum* infection, HaCaT keratinocytes modulate the expression of TLR2 [29] and TLR4 [30], which corroborates with our findings in co-culture assays. This condition mimics cutaneous-like infections and broadens the perspective that TLR genes are necessary for the host immune response in the first stages of infections. We also detected the differential expression of other TLR-coding genes not yet described as being involved in *T. rubrum* recognition by keratinocytes (Figure 1). Interestingly, TLR6, a receptor that drives the production of the anti-inflammatory IL-10 in *C. albicans* [31], was our study’s most abundant TLR transcript detected.

NLR family members (NOD-like receptors) are also a class of receptors widely studied in innate immune responses because they cooperate with TLRs to boost the immune response against pathogens [32,33]. Our results showed a positive modulation of the NOD1, NOD2, and NLRP3 genes after 24 h of co-culture (Figure 1), suggesting that in the early stages of infection, the transcription of these genes may be associated with the transcription of the TLR genes. This association would increase keratinocytes’ immune responses to combat fungal invasion. A role of NLRP3 in inflammasome formation is in interleukin-1β activation [26]. IL-1β production by bone-marrow-derived macrophages in response to *T. rubrum* seems to be NLRP3-dependent [34]. Therefore, we hypothesized that the transcriptional modulation of NLRP3 in the first three hours of infection is essential for subsequent IL-1β activation by the NLRP3 inflammasome. Recently, the secretion of IL-1β during the co-culture of HaCaT keratinocytes with *T. rubrum* has been reported [35].

*T. rubrum* has developed evasion mechanisms to escape or suppress the host immune response by inhibiting keratinocyte TLR expression [36]. β-glucans, an essential component of the fungal cell wall recognized by the Dectin receptors [37], are synthesized by glucan-synthases. Our results showed that the β-glucan synthase-coding genes (TERG_01127 and TERG_12108) (Figure 6) were downregulated in all instances of infection. These data agree with the non-differential expression of Dectin-1 observed in this study. This modulation profile did not occur in the presence of the keratin-containing-medium (Figure 7), suggesting that the presence of keratinocytes is a limiting factor to control glucan exposure in the cell wall. Notwithstanding this, a similar effect occurred in the expression pattern of the chitin-synthase genes (Figure 6). Despite this, the expression of TLRs was still upregulated in the host cells during co-culture, which might be related to the presence of other PAMPs (e.g., mannans-derived PAMPS) that were not evaluated in our study. Altogether, our findings raise the hypothesis that *T. rubrum* might adapt to the host challenge and remodel its cell wall to avoid host recognition, facilitating fungal surveillance and infection chronicity.

Glycolysis is critical in producing antifungal cytokines and fungicidal effector molecules in the host during fungal invasion [12]. In our work, several genes involved in glycolytic metabolism and nutrient signaling, such as GLUT1, PDHA, LDHA, HIF-1α, and MTOR, were differentially expressed during the fungal–host interaction. We detected a significant upregulation in the keratinocyte’s glucose transporter GLUT1 gene expression (Figure 2) and an increased intracellular glucose content uptake in co-culture assays (Figure 3A). This effect is relevant because glucose influx into immune cells fuels antimicrobial activity and enhances inflammatory responses, thereby directing immune activation and recruitment [12]. The transcription factor HIF-1α is directly involved in glucose metabolism by regulating the activity of glucose transporters, such as the GLUT1, and orchestrates immune cell activation toward an inflammatory state by promoting glycolysis, proinflammatory cytokine secretion, enhanced phagocytosis, and microbicide activity [38,39]. Thus, keratinocytes can respond to infections by activating the HIF-1α cascade to increase glucose uptake and subsequent glycolysis utilization, generating energy to fight the infection [14,39]. Previous studies suggest that mTOR signaling could influence GLUT1 expression by itself or via HIF-1α transcription [40,41]. Therefore, our results are consistent with the literature, as we observed a simultaneous increase in both HIF-1α and mTOR transcript levels after co-culture assays (Figure 2), which suggests that keratinocytes modulate the mTORC- HIF-1α cascade to fight fungal invasion through glucose metabolism. However, because of mTOR’s multifaceted roles, its differential expression during the interaction between HaCaT keratinocytes and *T. rubrum* prompts hypotheses regarding its importance in an infectious scenario, including its potential association with cell differentiation, the disruption of the host’s epithelial barrier caused by the fungal attack, and its involvement in the overexpression of GLUT1 and HIF-1α.

Under glucose metabolism conditions, the pyruvate dehydrogenase enzyme tends to convert glucose into pyruvate. Here, we show an upregulation of the pyruvate dehydrogenase gene (PDHA) at the initial stages of the co-culture assays, followed by a significant increase in lactate dehydrogenase (LDHA) gene expression (Figure 2). These results suggest that glycolysis substrates could be redirected to lactate production when the keratinocytes were challenged with fungal cells. This effect was confirmed when we measured lactate levels of co-culture supernatants at 24 and 48 h (Figure 4). Recent investigations have highlighted that lactate can function as an essential signaling molecule, exerting control over the effector functions of both innate and adaptive immune cells [42,43]. If glycolytic activation is accompanied by an inhibition of mitochondrial oxidative phosphorylation and increased lactate production, it results in a metabolic reorganization known as the Warburg effect. Such metabolic shifts are among the most common reprogramming events during species interactions [7].

Notwithstanding this, we also observed a decrease in the transcriptional response of the gene involved in mitochondrial oxidative phosphorylation (UQCC1) after 48 h of co-culture (Figure 2). However, whether a metabolic shift towards the Warburg Effect occurs in keratinocytes during the *T. rubrum* interaction cannot be determined based on only transcriptional data.

Our results showed that LDH activity in the cell supernatant was only observed at 24 h post-interaction. We did not detect LDH enzymatic activity in the cell culture supernatant after 48 h of co-culture (Figure 5B). Several factors can influence enzymatic activity, including pH changes. Considering the statistics of pH monitoring, there is a tendency towards an acidification of the cultivation medium with or without co-cultivation. Meanwhile, the release of lactate in the co-culture tends to acidify the medium. This observation indicated an effect of pH on the secretion of enzymes and proteins or even a strong influence on their activity.

Cells release LDH when there is damage to the cell membrane, and measuring LDH release indicates cell viability. In a prior study, the percentage of LDH release ranged from 30% to 45% after 24 h and increased from 60% to 70% after 48 h when HaCaT keratinocytes were co-cultured with *T. rubrum* [35]. Here, we observed that tissue monolayers were whole despite the fungal invasion expected in co-culture assays after 48 of co-culture, showing that keratinocytes were viable during this time (Figure S1).

**Figure 8:**
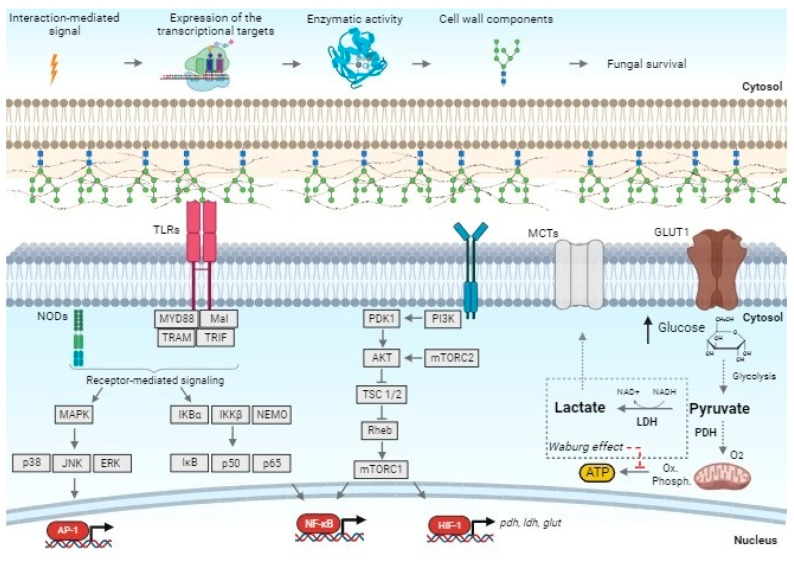
Proposed model of early host–pathogen interaction focusing on the signaling of human keratinocytes co-cultured with *T. rubrum*. Upon fungal recognition by keratinocytes, immunological cascades are triggered to prevent fungal colonization. TLRs mediate the recognition of fungal PAMPs and activate transcription responses enacted by AP-1 and NF-κB. At the same time, the PI3K/Akt/mTOR pathway controls glucose metabolism, which also results in NF-κB recruitment. In addition, HIF-1α plays a role in pyruvate dehydrogenase (PHDA), lactate dehydrogenase (*LDHA*), and glucose transporter (*glut*) expressions. The Warburg effect might also result from glucose metabolism after fungal interaction with HaCaT cells, through the monocarboxylate transporter MCL in the keratinocyte membrane. Fungus contacting HaCaT cells triggers the expression of enzymes involved in the biosynthesis of cell wall components. We have created this figure with the help of BioRender.com.

Briefly, our findings reveal how keratinocytes can modulate and adapt their immunological and metabolic transcriptional programs to respond to and fight *T. rubrum* invasion. We suggest that TLRs are important for *T. rubrum* recognition and that this will trigger fungal cell wall remodeling to evade the host immune response. Altogether, our transcriptional data suggest that metabolic adaptability is critical in controlling and overcoming infections by metabolizing and directing glucose to energy and sub-product generation. Further investigations to better comprehend these results include the exploration of a standard keratinocyte cell line, a skin model, and other dermatophyte species. Figure 8 shows a proposed model for early host–pathogen interaction, containing our main findings, perspectives, and hypotheses for future research.

## 5. Conclusions

Our results reveal the differential transcription regulation of genes encoding PRRs during an infection-like scenario. Furthermore, we demonstrated variations in the transcriptional balance of genes related to the cell wall when the fungus was exposed to keratinocytes or a keratin-containing medium. These findings suggest a potential mechanism of fungal evasion to hinder host recognition, thereby facilitating pathogen dissemination through the stratum corneum during infection. Notably, we showed that essential genes involved in glycolytic metabolism are overexpressed during the interaction between *T. rubrum* and keratinocytes. This overexpression is accompanied by increased glucose and lactate levels during co-culture. This phenomenon implies that metabolic adaptability towards glycolytic metabolism could enhance the immunological host response against *T. rubrum*.

## Figures and Tables

**Table 1:** pH analysis of the cell culture supernatant during the co-culture of HaCaT cells with *T. rubrum*.

Time	HaCat	Co-Culture
3 h	6.8 ± 0.15	7.2 ± 0.3
24 h	7.2 ± 0.3	7.1 ± 0.5
48 h	6.9 ± 0.5	6.5 ± 0.41

## Data Availability

Data are contained within the article and supplementary materials.

## Supplementary Materials

The following supporting information can be downloaded at: https://www.mdpi.com/article/10.3390/jof10010072/s1, Figure S1: Optical microscopy of co-culture assay in 3 h, 24 h, and 48 h. Table S1: Primers used for RT-qPCR analysis.
