# Supplementary material for: Molecular Signaling and Metabolic Responses during the Interaction between Human Keratinocytes (HaCaT) and the Dermatophyte Trichophyton rubrum
Source: J Fungi (Basel). 2024 Jan 16;10(1):72. doi: 10.3390/jof10010072 (PMC10820588; doi:10.3390/jof10010072)
Supplement: Supplementary file 1 [file jof-10-00072-s001.zip › jof-2695959-supplementary.pdf]

Supplementary Material

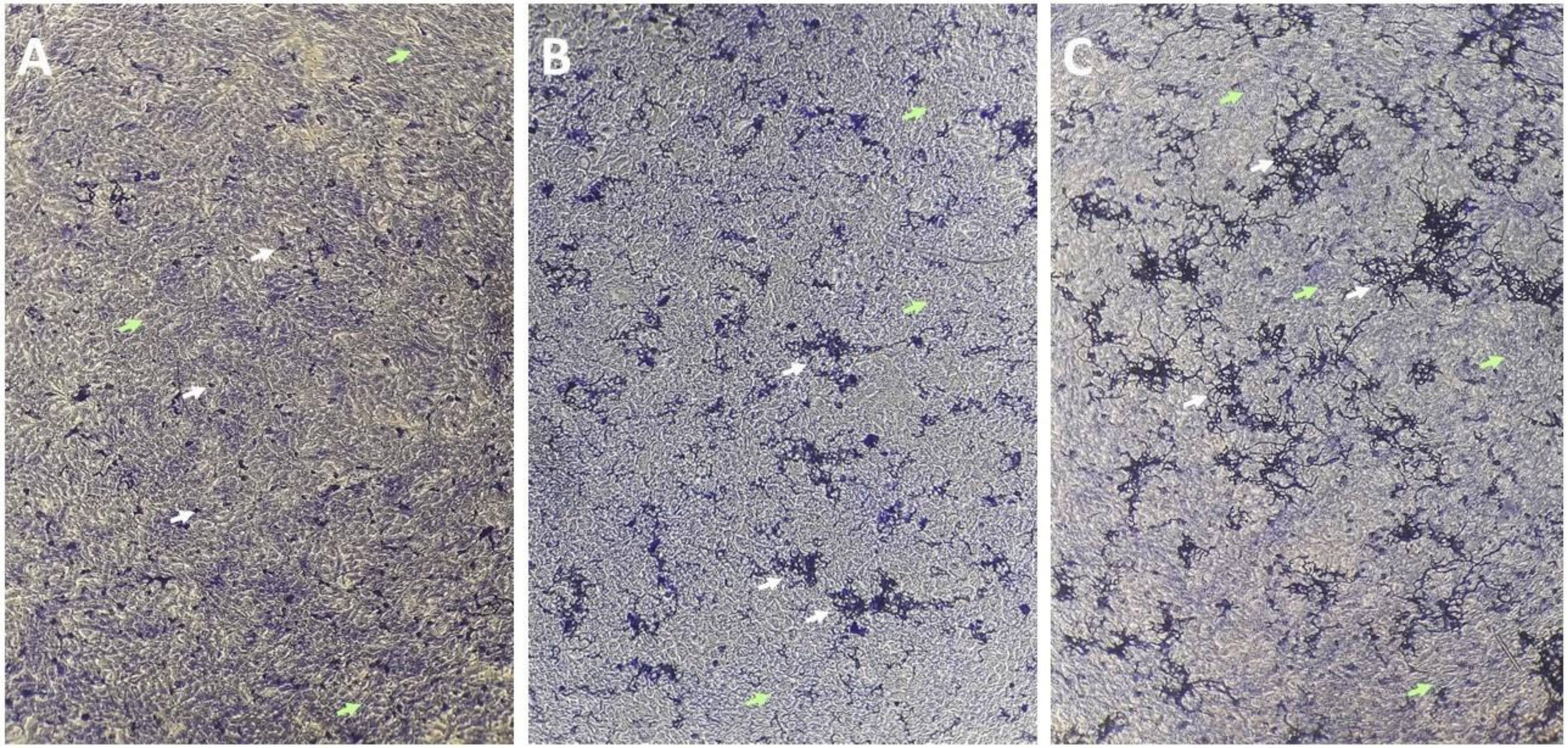

Figure S1. Optical microscopy of HaCaT keratinocytes co-cultured with *Trichophyton rubrum* stained with May Grunwald-Giemsa for 3 hours (A), 24 hours (B), and 48 hours (C). White arrows indicate fungal fragments adherent to the cells, and green arrows show the intact keratinocyte monolayer cells adhered to the culture plate.

Table S1. Primer sequences used for RT-qPCR analysis.

Gene ID	GenBank Accession	Gene Product Name	Primer sequence (5′-3′)	Amplicon
TLR1	NM_003263	Toll-like receptor 1	F: CCGGAAAGTTATAGAGGAACCC<br>R: CAGATCCAAGTAGCTGCAGAG	144 bp
TLR2	NM_003264	Toll-like receptor 2	F: ATCCTCCAATCAGGCTTCTCT<br>R: GGACAGGTCAAGGCTTTTTACA	118 bp
TLR4	NM_138557	Toll-like receptor 4	F: TCTACAAAATCCCCGACAACC<br>R: TGTCTGGATTTCACACCTGG	143 bp
TLR5	NM_003268	Toll-like receptor 5	F: AAACATGGTGCTGGAGATACTAG<br>R: GTGGGCAAGAATCAAAGAGAAG	115 bp
TLR6	NM_006068	Toll-like receptor 6	F: CCAGAAAGTTATAGAGGAAGCCC<br>R: CAGATCCAAGTAGATGCAGAGG	144 bp
NOD1	NM_006092	Nucleotide binding oligomerization domain containing 1	F: AAGTGATTCTGTCCCTCCAAAG<br>R: CCCGTTTAGTCACCCTTCAG	143 bp

NOD2	NM_022162	Nucleotide binding oligomerization domain containing 2	F: CCCTGCTCTTCAACCTTCTG<br>R: CTGTTCAGAGAAGCCCTTGAG	137 bp
NLRP3	NM_001127462	NLR family pyrin domain containing 3	F: CCACAAGATCGTGAGAAAACCC<br>R: CGGTCCTATGTGCTCGTCA	91 bp
HUMAN GAPDH	NM_001256799	Glyceraldehyde-3-Phosphate dehydrogenase	F: AATCCCATCACCATCTTCCAG<br>R: GAGCCCCAGCCTTCTCCAT	118 bp
HUMAN BETA-ACTIN	DQ407611.1	Beta-actin	F: GTTGCGTTACACCCTTTCTTG<br>R: TGCTGTCACCTTCACCGTTC	154 bp
MTOR	NM_004958	Mechanistic target of rapamycin kinase	F: GCAGATTTGCCAACTATCTTCGG<br>R: CAGCGGTAAAAGTGTCCCCTG	114 bp
HIF-1α	NM_001530.4	Hypoxia inducible factor 1 subunit alpha	F: GAACGTCGAAAAGAAAAGTCTCG<br>R: CCTTATCAAGATGCGAACTCACA	124 bp
GLUT1	NM_006516.4	Glucose transporter 1	F: TCTGGCATCAACGCTGTCTTC<br>R: CGATACCGGAGCCAATGGT	94 bp

PDHA	NM_000284.4	Pyruvate dehydrogenase E1 alpha subunit	F: ATGGAATGGGAACGTCTGTTG<br>R: CCTCTCGGACGCACAGGATA	114 bp
LDHA	NM_001165415	Lactate dehydrogenase A	F: TTGACCTACGTGGCTTGGAAG<br>R: GGTAACGGAATCGGGCTGAAT	91 bp
UQCC1	NM_018244.5	Ubiquinol-cytochrome c reductase complex assembly factor 1	F: GGAGAAAACTGACTTCGAGGAAT<br>R: TCCAGACGTGGAGTAGGGTTA	92 bp
TERG_01127	XM_047749245.1	1,3-beta-glucan synthase component FKS1	F: TTGACTGAGCGTGGTTTCTG<br>R: GGCGTAGATCTGACACACGA	99 bp
TERG_12108	XM_047751115.1	1,4-alpha-glucan-branching enzyme	F: CCTCACCAACCGTAGACATG<br>R: CACCGACAAGCCTATCCATC	137 bp
TERG_03843	XM_047749656.1	Chitin synthase B	F: CTGAAGTCTGTATCCTGCTCG<br>R: TCCTTTACCCAACATAGCGTG	134 bp
TERG_02562	XM_047749438.1	Chitin synthase C	F: CCTCAACATCTACGCCTTCTG<br>R: CGTTAAGATCTCCGTCATCCTG	146 bp

TERG_12319	XM_003233728.2	Chitin synthase 2	F: AGCCAACTGCCTTGTACCAT<br>R: GTAATCCGACCCATCCCTTT	108 bp
rpb2	XM_003235036.2	DNA-directed RNA polymerase II core subunit RPB2	F: TGCAGGAGGTTTGATGAAGA<br>R: GCTGGGAGGTACTGTTTGATCAA	59 bp
gapdh	XM_047749776.1	Glyceraldehyde-3-phosphate dehydrogenase	F: GCGTGACCCAGCGATGTAGT<br>R: CGGTGGACTCGACGATGTAGT	62 bp
